# Circadian pattern of short-term variability of the QT-interval in primary prevention ICD patients - EU-CERT-ICD methodological pilot study

**DOI:** 10.1371/journal.pone.0183199

**Published:** 2017-08-21

**Authors:** David J. Sprenkeler, Anton E. Tuinenburg, Henk J. Ritsema van Eck, Marek Malik, Markus Zabel, Marc A. Vos

**Affiliations:** 1 Department of Medical Physiology, Division of Heart and Lungs, University Medical Center Utrecht, Utrecht, The Netherlands; 2 Department of Cardiology, Division of Heart and Lungs, University Medical Center Utrecht, Utrecht, The Netherlands; 3 Department of Medical Informatics, Erasmus University Medical Center, Rotterdam, The Netherlands; 4 National Heart & Lung Institute, Imperial College London, London, United Kingdom; 5 Department of Cardiology and Pneumology, Division of Clinical Electrophysiology, University Medical Center Göttingen, Göttingen, Germany; University of Adelaide, AUSTRALIA

## Abstract

**Objective:**

Short-term variability of the QT-interval (STV-QT) was shown to be associated with an increased risk of ventricular arrhythmias. We aimed at investigating (a) whether STV-QT exhibits circadian pattern, and (b) whether such pattern differs between patients with high and low arrhythmia risk.

**Methods:**

As part of the ongoing EU-CERT-ICD study, 24h high resolution digital ambulatory 12-lead Holter recordings are collected prior to ICD implantation for primary prophylactic indication. Presently available patients were categorized based on their arrhythmia score (AS), a custom-made weighted score of the number of arrhythmic events on the recording. STV-QT was calculated every hour in 30 patients of which 15 and 15 patients had a high and a low AS, respectively.

**Results:**

The overall dynamicity of STV-QT showed high intra- and inter-individual variability with different circadian patterns associated with low and high AS. High AS patients showed a prominent peak both at 08:00 and 18:00. At these times, STV-QT was significantly higher in the high AS patients compared to the low AS patients (1.22ms±0.55ms vs 0.60ms±0.24ms at 08:00 and 1.12ms±0.39ms vs 0.64ms±0.29ms at 18:00, both p < 0.01).

**Conclusion:**

In patients with high AS, STV-QT peaks in the early morning and late afternoon. This potentially reflects increased arrhythmia risk at these times. Prospective STV-QT determination at these times might thus be more sensitive to identify patients at high risk of ventricular arrhythmias.

## Introduction

Sudden cardiac death (SCD) due to ventricular arrhythmias remains an important health problem, accounting for approximately 60% of total cardiovascular mortality.[1] Prophylactic implantation of implantable cardioverter-defibrillators (ICD) was shown to reduce mortality in patients with a reduced left ventricular ejection fraction (LVEF).[2,3] LVEF reduction is presently used in international guidelines as a class I indication for ICD implantation in patients with left ventricular dysfunction.[4] However, LVEF lacks sensitivity and specificity of patients stratification, since a large proportion of patients suffering from sudden cardiac arrest have LVEF above the 35% cut-off.[5] Furthermore, in recent registries of ICD recipients, the incidence of appropriate shock [6,7] was found substantially lower than that reported in the original MADIT II and SCD-HeFT trials,[2,3] possibly explained by improved pharmacological heart failure treatment and ICD-programming algorithms. Consequently, up to two thirds of ICD recipients will never receive an appropriate ICD shock during their lives,[8] indicating that more accurate characterization of a patient at high SCD risk is urgently needed. In the search for better risk assessment, ECG-derived risk parameters are continuously investigated since the ECG is an inexpensive, easy to use, and widely accessible non-invasive tool.

Short-term variability of the QT-interval (STV-QT) is a relatively new ECG-based parameter that captures the repolarization instability in 30 consecutive beats.[9] An increased STV-QT was shown in patients with drug-induced and congenital long QT-syndrome and in patients with non-ischemic heart failure with a history of ventricular arrhythmias.[10–12] Presently, the predictive value of STV-QT is further evaluated in the primary prevention ICD-population as part of the EUropean Comparative Effectiveness Research to assess the use of primary prophylacTic Implantable Cardioverter Defibrillators (EU-CERT-ICD) study. In contrast to previous studies in which STV-QT was measured in 2-minute 12-lead ECG recordings, the EU-CERT-ICD study uses high resolution 24-hour Holter recordings for STV-QT analysis. The use of long-term recordings leads to the question of when to take a 30-beat sample during the 24 hours of the recording.

Yet, little is known about the STV-QT changes during the day and/or whether the circadian pattern of STV-QT is different in patients at low and high SCD risk. If such a difference exists, calculation of STV-QT at a certain time of the day might increase the sensitivity and specificity of this parameter in identifying the patients at risk. Therefore, as a methodological pilot study of EU-CERT-ICD, we aimed at investigating (a) whether STV-QT exhibits a circadian pattern and (b) whether this pattern differs between high and low risk patients.

## Materials & methods

### Study design

The EU-CERT-ICD study is a currently enrolling prospective, multicenter, observational study (NCT 02064192) that aims to assess electrocardiographic parameters for prediction of all-cause mortality and appropriate ICD-shocks. The study plans to recruit 2500 patients with ischemic and non-ischemic cardiomyopathy fulfilling the international treatment guidelines criteria for primary prophylactic ICD implantation.[13] Patients who are candidate for cardiac resynchronization therapy (CRT) or secondary prophylactic ICD are excluded. The protocol was approved by the institutional review board or ethics committee at each participating hospital and was in compliance with the Declaration of Helsinki. All patients provided written informed consent.

Clinical characteristics including age, sex, race, NYHA class, comorbidities and cardiovascular drug treatment are collected at baseline. Prior to ICD implantation, all patients also undergo a 24-hour 12-lead digital Holter recording using the SEER 12 recorder programmed at 1024 Hz sampling frequency (Getemed, Teltow, Germany). In addition to STV-QT, other ECG-derived parameters, including microvolt T-wave alternans, heart rate variability, heart rate turbulence, and T-wave morphology will be prospectively assessed for the risk stratification purposes. In the current methodological pilot study, STV-QT of 30 cardiac cycles was determined at the beginning of every hour during the 24h recording in a subpopulation of already enrolled patients.

### Measurement of STV-QT

STV-QT was determined in lead V2 and was calculated using the method of fiducial segment averaging (FSA).[14] First, each QRS-complex was aligned around a trigger point (usually the the R-peak) by cross correlating each individual complex with the average of the other complexes and then shifted until maximal correlation is achieved. Next, the different fiducial points, i.e. the QRS-onset and the end of T-wave, were aligned separately by the same technique using a segment of 30 samples around the fiducial point. Correct alignment was checked visually by one of the authors (D.S.) and manually adjusted where necessary. The advantage of our custom-made software is that the program preserves the amount of shifting for each individual beat and thus the QT-interval of each beat can be derived from the individual fiducial point estimates.

Using Poincaré plots, the QT-interval of each complex was plotted against the former. STV-QT was defined (as proposed by Thomson et al. [15]) as the mean orthogonal distance of the points to the line of identity, calculated by the formula $\sum |D_{n+1}-D_{n}|/N\times \sqrt{2}$, where D represents the QT-interval and N is the number of beats (30 in this case). Ventricular and atrial premature complexes together with the following post-extrasystolic beat were excluded from the analysis. In addition to STV-QT, both the RR-interval and QT-interval of the 30 beats were measured automatically.

### Endpoints

No definite endpoints, e.g. all-cause mortality or appropriate ICD shocks, are presently known. Nevertheless, previous studies have shown that the presence of non-sustained ventricular tachycardia (nsVT) and/or a high frequency of premature ventricular complexes (PVC) are independent predictors of SCD and/or appropriate ICD shocks.[16,17] Therefore, to differentiate between high and low risk patients, the number of arrhythmic events on the Holter recording was used as a surrogate endpoint. A custom-made arrhythmia score (AS) was designed for this purpose and described in Table 1. Arbitrarily and solely for the purposes of this methodological pilot study, patients were classified based on their AS into three groups: low AS (< 100 points/24h), moderate AS (between 100 and 1000 points/24h) and high AS (>1000 points/24h). Only low AS and high AS patients were included in the present methodological study in order to differentiate clearly between low and high arrhythmia risk.

**Table 1:** Arrhythmia score (AS).

Arrhythmic event	points
**Single PVC**	1
**Couplet**	2
**Triplet**	3
**Bigeminy**	4
**Non-sustained VT (> 3 complexes)**	5

AS is defined as the sum of the points per 24 hours

### Statistical analysis

Data are expressed as mean ± standard error of the mean (SEM). Repeated ANOVA with Bonferroni correction for multiple comparisons was used for within group analysis. Between-group analysis was performed using two-tailed Student t-test assuming different variances. Pearson’s correlation coefficients were used for correlation analyses. Calculations were performed using SPSS (version 23, IBM). P-values ≤ 0.05 were considered significant.

## Results

Out of the EU-CERT-ICD Holter database, AS was assessed for already enrolled patients. Patients with atrial fibrillation, less then 23 hours of noise-free recording or flat T-waves in the precordial leads were excluded. A total of 30 patients were selected based on their AS, of which 15 and 15 patients had a high and low AS, respectively.

### Baseline criteria

Table 2 shows baseline characteristics of the patients included in the analysis. The mean age was 60 ± 2 years and 80% were men. Patients with high AS appeared to be slightly but not significantly older compared to the low AS patients (p = 0.06). A slight majority of patients had ischemic cardiomyopathy (53%) with a mean left ventricular ejection fraction of 27% ± 1%. Significantly more patients in the high AS group had hypertension. The use of heart failure medications was not different between the two groups.

**Table 2:** Baseline characteristics of study cohort (n = 30).

Mean ± SEM or N (%)	low AS (n = 15)	high AS (n = 15)	Total (n = 30)
**Age,** years	55.8 ± 3.4	64.3 ± 2.4	60.1 ± 2.2
**Sex**			
*Female*	3 (20%)	2 (13.3%)	5 (17%)
*Male*	12 (80%)	13 (86.7%)	25 (83)
**Leading cardiac disease**			
*DCM*	7 (46.7%)	6 (40%)	13 (43%)
*ICM*	8 (53.3%)	9 (60%)	17 (57%)
**LVEF,** percentage	26.8 ± 1.7	27.0 ± 1.7	26.9 ± 1.2
**NYHA**			
*I or II*	11 (73%)	9 (60%)	20 (67%)
*III*	4 (27%)	6 (40%)	10 (33%)
**Smoking**	11 (73.7%)	10 (66.7%)	21 (70%)
**Diabetes mellitus**	5 (33.3%)	7 (46.7%)	12 (40%)
**hypertension**	4 (26.7%)	13 (86.7%)*	17 (57%)
**Beta-blocker**	14 (93.3%)	14 (93.3%)	28 (93%)
**ACEi/ARB**	15 (100%)	13 (86.7%)	28 (93%)
**MRA**	12 (80%)	15 (100%)	27 (90%)
**statin**	12 (80%)	11 (73.3%)	23 (77%)
**Class I or III antiarrhythmic drugs**	1 (6.7%)	2 (13.3%)	3 (10%)

* p < 0.05 high AS vs low AS. AS = arrhythmia score; DCM = dilated cardiomyopathy; ICM = ischemic cardiomyopathy; NYHA = New York Heart Association; ACEi = ACE-inhibitor; ARB = angiotensin II receptor blocker; MRA = mineralocorticoid receptor antagonist.

### Diurnal pattern of RR and QT

**Fig 1:**
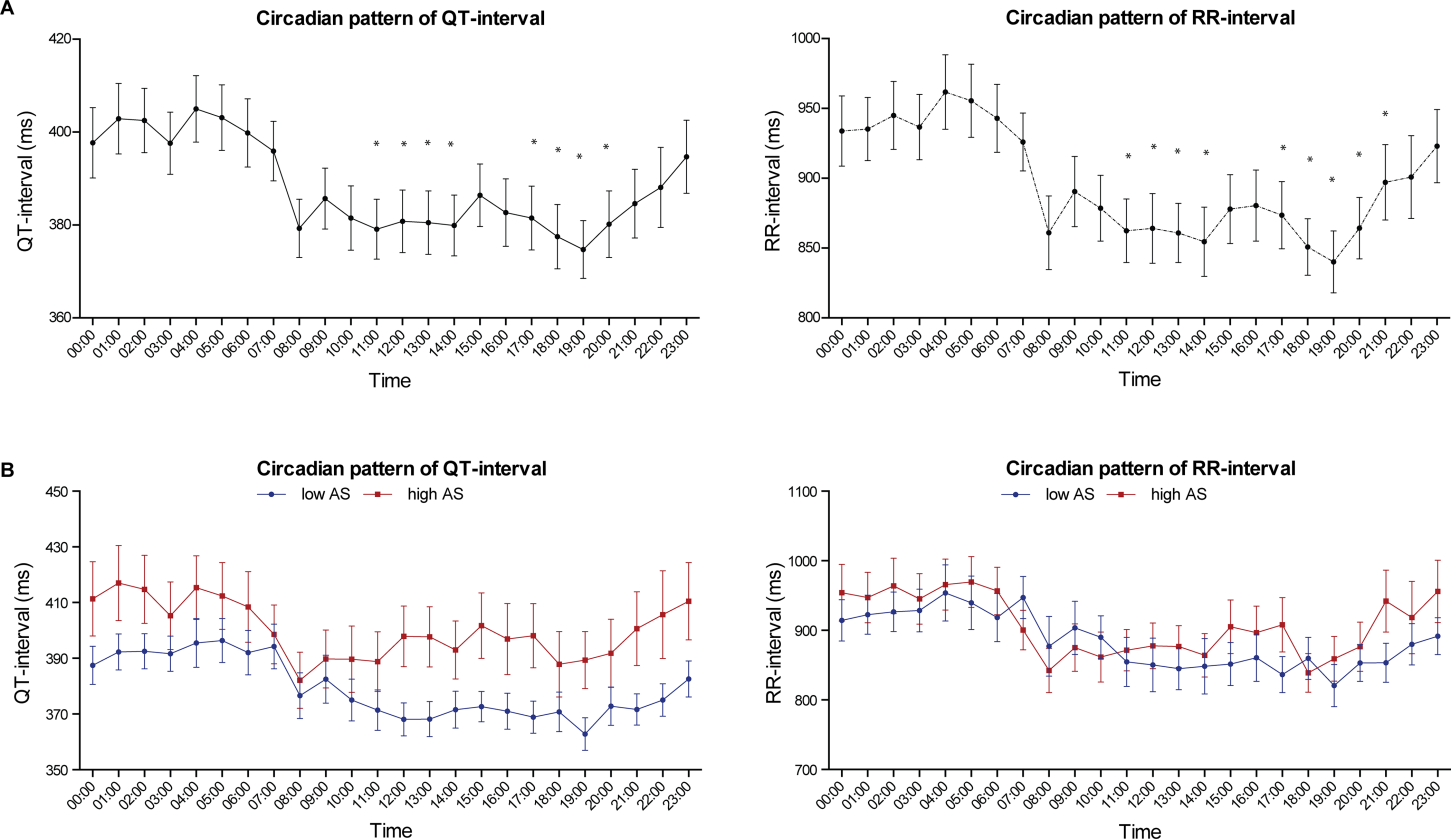
Circadian pattern of RR- and QT-interval. **(**A) mean ± SEM at beginning of every hour of total cohort (n = 30). Significant higher values are seen at night compared to during the day. * = p< 0.05 compared to 0:00. (B) Mean ± SEM at beginning of every hour of low AS-group (blue line, n = 15) and high AS group (red line, n = 15). No significant differences are found in the circadian pattern of RR-interval or QT-interval between low and high AS group.

The circadian profile of RR- and QT-intervals is shown in Fig 1. As expected, both show higher values at night with a peak at around 04:00, a clear drop in the morning and significantly lower values during the day. No significant differences were found in RR-interval between the low AS and high AS group (Fig 1B). The QT-interval of the high AS appeared to be slightly but not significantly longer than that of the low AS group during the day.

### Diurnal pattern of STV-QT

**Fig 2:**
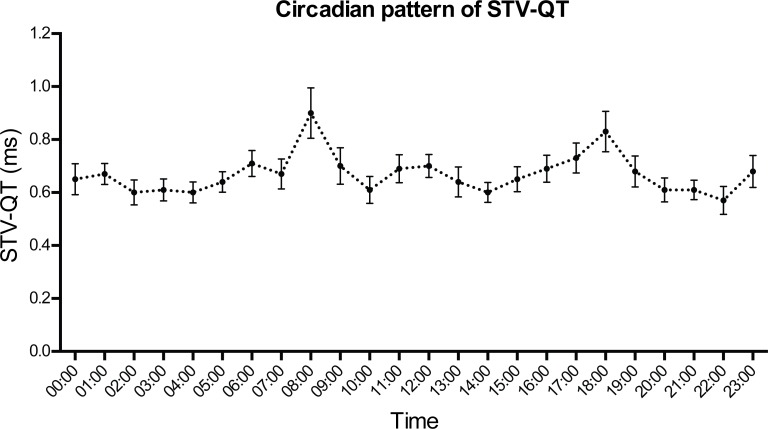
Circadian pattern of STV-QT. Mean ± SEM at beginning of every hour of total cohort (n = 30). No clear circadian pattern is found, however, two non-significant peaks at 08:00 and 18:00 can be discerned.

**Fig 3:**
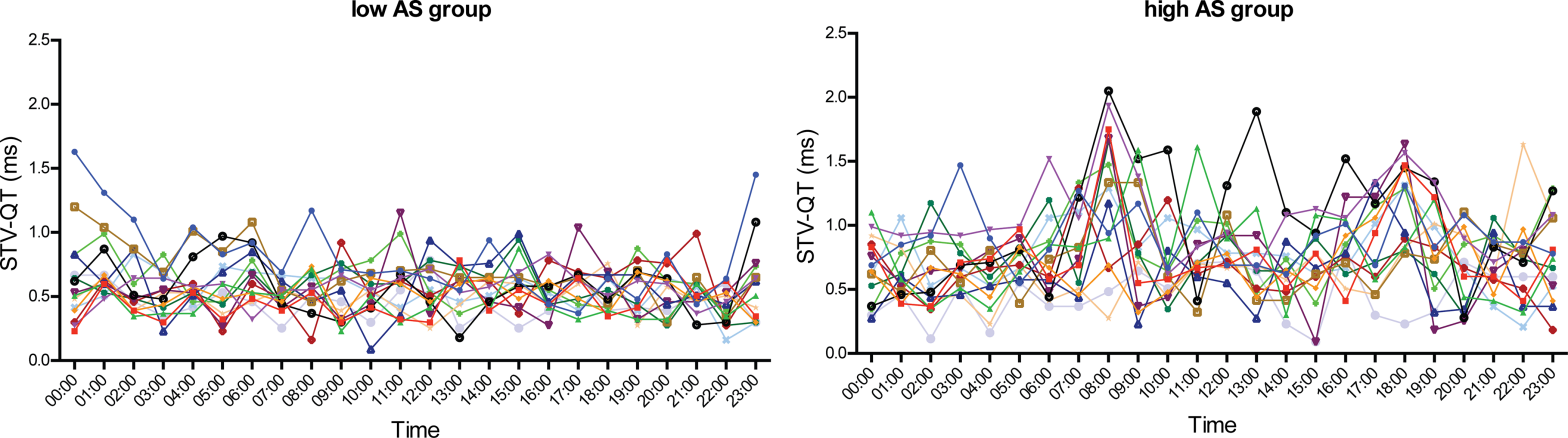
Circadian pattern of STV-QT in individual patients. Individual patients in low AS group (left) and high AS group (right). High inter- and intraindividual variability can be seen, which is more pronounced in the high AS group.

**Fig 4:**
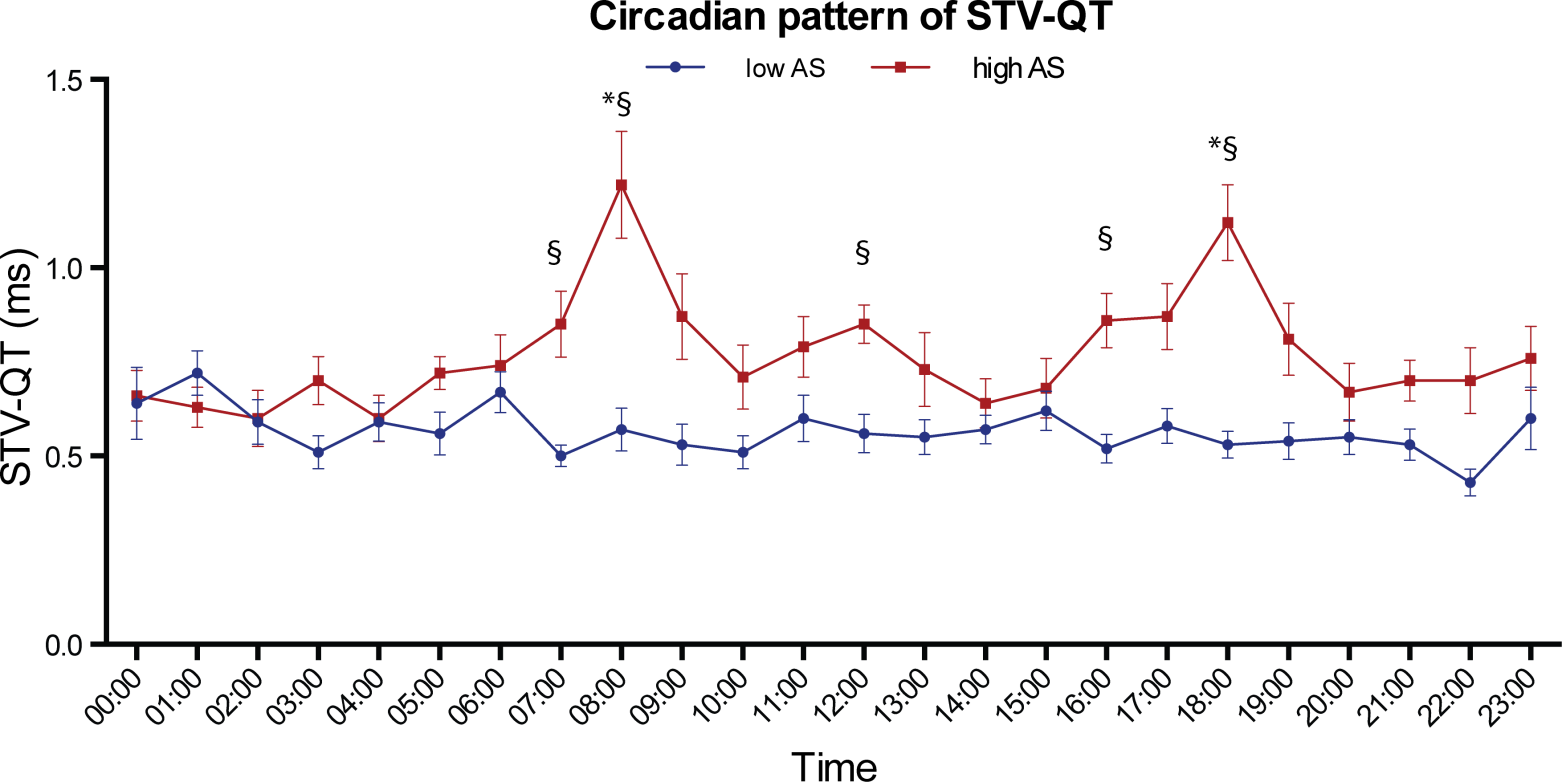
Circadian pattern of STV-QT in AS subgroups. Mean ± SEM at beginning of every hour in low AS (blue line, n = 15) and patients with high AS (red line, n = 15). * p < 0.05 compared to 0:00; § p <0.05 compared to low AS. STV-QT peaks at 08:00 and 18:00 in high AS patients, but is stable during the day in low AS patients.

No clear circadian pattern of STV-QT was found in the total cohort. Nevertheless, two small non-significant peaks are visible at 08:00 and 18:00 (Fig 2). A high intra- and inter-individual variability of STV-QT was seen, mainly in the high AS group (Fig 3). A different hour-by-hour behaviour of STV-QT was seen in the low AS-group and high AS-group (Fig 4). While the low AS patients showed a more-or-less stable STV-QT during the day with low variability, high AS patients showed significant STV-QT peaks (p < 0.05) at 08:00 and 18:00. At these time points, STV-QT was significantly higher in high AS patients compared to low AS patients (1.22ms±0.55ms vs 0.60ms±0.24ms at 08:00 and 1.12ms±0.39ms vs 0.64ms±0.29ms at 18:00, both p < 0.01). At other time points, 07:00, 12:00 and 16:00, STV-QT was also increased in the high AS group, but the differences were less expressed. At several time points, especially during the night, STV-QT did not differ between the low and high AS groups.

## Discussion

The presented results show (a) STV-QT intra- and interindividual variability, and (b) distinct STV-QT peaks in the early morning and late afternoon that are not seen in the low AS patients. Interestingly, at both of these time points the QT interval was the shortest. In previous animal studies, a positive relation was found between STV and action potential durations (APD) with higher STV at longer APD.[18] This can be explained by the absolute increase in APD variation at longer APD and does not reflect higher repolarization instability. Nevertheless, the opposite was found in the high AS patients in the current study: the highest STV-QT was seen when QT-interval was the shortest. This implies that an independent proarrhythmic component might be partly responsible for this STV-QT increase.

In previous STV-QT clinical studies, a 2-minute ECG was taken at arbitrary during day-time hours. Nevertheless, as we have shown, the discriminative power of STV-QT varies substantially between 8.00 and 17:00. For instance, in the data of this patient sub-population, samples taken around 14:00 or 15:00 would be less useful for the distinction between the low and high AS groups. It seems plausible to propose that sampling at the time points of 8:00 or 18:00 might increase the predictive capabilities of STV-QT.

### STV-QT as a marker of arrhythmic risk

Finding a highly sensitive and specific ECG-derived parameter predictive for sudden cardiac death remains the holy grail in the field of electrocardiology. In particular, a risk stratification technique is urgently needed that identifies arrhythmic rather than overall mortality risk. The most promising ECG-based risk parameters, such as microvolt T-wave alternans [19] or QT-variability index (QTVI)[20] are markers of abnormal repolarization and reflect the electrical substrate that predisposes to ventricular arrhythmias. However, none of these parameters have yet been incorporated into clinical practice. STV-QT has the advantage to reflect instability of repolarization on a consecutive basis and can be measured on 30 complexes instead of 256 complexes that are required to calculate QTVI. The method of fiducial segment averaging eliminates the problem of identification of the end of the T-wave of every complex separately, therefore diminishes measurement error.[14] STV-QT has been investigated in a number of retrospective studies with a wide variety of populations that are at risk of SCD and shows potential for arrhythmic risk prediction. As part of the EU-CERT-ICD study this parameter will be evaluated prospectively in a large cohort of primary prophylactic ICD patients to further establish its role in risk stratification.

### Circadian pattern of STV-QT resembles circadian profile of sudden cardiac death

Interestingly, the distinct peaks of STV-QT in the early morning and late afternoon in the high AS patients resemble the circadian distribution of sudden cardiac death found in large population-based studies.[21,22] These large studies found a peak between 6:00 and noon and a secondary lower peak between 17:00 and 18:00. The same diurnal distribution was found in studies investigating the circadian variation of appropriate ICD-shocks.[23,24] The increased incidence of SCD in the morning may be linked to arousal-related increase of the sympathetic tone, which might increases the myocardial electrical instability and decreases the threshold for ventricular fibrillation. In experimental studies, left stellate ganglion nerve activity (SGNA), which reflects discharge of the sympathetic nervous system, was reported to show similar circadian profile with a high peak in the early morning in dogs with pacing induced heart failure or experimentally induced myocardial infarction.[25–27] SGNA increase was also shown to precede the occurrence of ventricular arrhythmia.[28]

### QT variability and the autonomous nervous system

A relation between autonomic tone and higher beat-to-beat QT variability has previously been reported. Yeragani et al investigated the effect of posture and isoproterenol infusion on QT variability and described significantly higher QT variability in standing position and after the infusion of isoproterenol.[29] Recently, a significant correlation was shown between elevated STV-QT and parameters of sympathetic predominance in patients with impaired glucose tolerance.[30] A study by Piccirillo et al. found a positive relation between the level of anxiety and a high QTVI.[31] On the other hand, QT variability was significantly reduced after administration of metoprolol or carvedilol compared to placebo in patients with ischemic cardiomyopathy.[32] Also, in a SGNA activity study, the dogs with high sympathetic activity showed significantly higher QT variability index compared to dogs with low sympathetic activity.[33] Noteworthy, this difference was only seen in the dogs with pacing-induced heart failure. Although all these studies used other QT-variability expressions, it seems reasonable to expect the same differences for STV-QT.

The increased beat-to-beat repolarization variability during high adrenergic drive might be caused by the autonomous nervous system effects during reduced repolarization reserve. In cellular experiments under baseline conditions, selective I_Ks_ blockade does neither increase repolarization variability nor results in arrhythmias because of compensatory effects of other repolarizing currents[34]. However, when β-adrenergic stimulation is added to I_Ks_ blockade, repolarization variability increases significantly and early and delayed afterdepolarizations start to occur.

In our study population, i.e. patients with left ventricular dysfunction, a reduced repolarization reserve is likely because of repolarizing currents downregulation.[35] Sympathetic activity during arousal might thus exhaust the repolarization reserve explaining the STV-QT rise. The reasons for the second peak in the late afternoon are more speculative. Its might be related to periprandial changes in the autonomic modulations.

### Limitations

The present study has important limitations. First, because of nature of a methodological pilot study, the sample size is small. Second, we used a custom-made score of the number of arrhythmic events on the Holter recording to categorize patients into high and low arrhythmic risk. Whilst this might reasonably correspond to some previous publications, we have no data on actual arrhythmic risk in these patients. A recent study by Seegers et al[16] found a high number of premature ventricular complexes predicting appropriate ICD shocks but not all-cause mortality. Nevertheless, in a substudy of the Prospective Randomized Milrinone Survival Evaluation (PROMISE) trial, high burden of ventricular ectopy on Holter recordings predicted SCD.[17] Finally, no continuous measurement of STV-QT during the 24 hours was performed. The current method of FSA is semi-automatically which requires manual identification of fiducial points. Currently, a fully automatic version is being developed and the first results show accurate STV-QT measurements in simulated data.[36]

## Conclusions

STV-QT shows high intra- and inter-individual 24-hour variability with peaks in the early morning and late afternoon in high AS patients. Studies on the circadian variation of SCD show a similar circadian profile. Determination of STV-QT at these time point might be more sensitive in identifying the patient at risk. It is therefore plausible to propose that the evaluation of the predictive power of STV-QT in the EU-CERT-ICD study concentrates primarily on these time points.

## Supporting information

S1 File The used data set is publicly available via PLOS ONE. (XLSX)
